# Molecular Detection and Seroprevalence Study of Peste des Petits Ruminants Virus in Small Ruminants in Central Ethiopia

**DOI:** 10.1155/vmi/9956146

**Published:** 2026-04-26

**Authors:** Teshome Yonas, Mishamo Sulayeman, Kassaye Adamu

**Affiliations:** ^1^ Jinka University, Jinka, Ethiopia; ^2^ Faculty of Veterinary Medicine, Hawassa University, P.O. Box 05, Hawassa, Ethiopia, hu.edu.et; ^3^ National Veterinary Institute, P.O. Box 19, Bishoftu, Ethiopia, sva.se

**Keywords:** C-ELISA, PPRV isolation, risk factors, RT–PCR, Vero cell culture

## Abstract

**Background:**

Peste des petits ruminants (PPR) is a severe, highly contagious, and fatal viral disease of small ruminants that causes significant production losses and mortality in Ethiopia. A cross‐sectional study was conducted from December 2021 to July 2022 to determine the seroprevalence of the disease in the study area. Samples were collected from the outbreak to conduct viral isolation and molecular detection, focusing specifically on sheep and goats as the study animals in the investigation.

**Materials and Methods:**

Clinical specimens were first collected from small ruminants showing signs suggestive of PPR. Paired swab samples and postmortem tissue specimens were obtained for virus isolation and molecular characterization. During necropsy, representative tissue sections were aseptically trimmed and processed. Of 250 clinically suspected animals, 25 were selected for detailed virus isolation and molecular detection of PPR virus (PPRV). PCR‐positive samples were further confirmed through virus isolation in cell culture. Subsequently, blood samples (4 mL) were collected from the jugular vein of 384 small ruminants older than 6 months for serological analysis. Serum was separated and analyzed to determine the seroprevalence of the infection.

**Results:**

PPRV was detected in 10 of 25 samples (40%)using reverse transcriptase PCR (RT–PCR), and of these RT–PCR‐positive samples, PPRV was isolated from four samples using Vero cells. The overall seroprevalence of PPRV was 20.6% (*n* = 384). Age, herd composition, and history of the recent introduction of new small ruminants were significantly associated with seropositivity of PPR (*p* < 0.05).

**Conclusion:**

The study confirmed widespread circulation of PPRV in the study areas, significantly affecting small ruminant productivity. High seroprevalence indicated endemicity, with identified risk factors including introducing new small ruminants and herd composition. Recommendations emphasize integrating small ruminants with other livestock and supporting government efforts. Caution is needed due to regional variations, highlighting the importance of further molecular characterization of circulating PPRV strains.

## 1. Introduction

Livestock is a major source of agricultural gross domestic product (GDP) in Ethiopia, contributing nearly 20% of the total GDP and 20% of national foreign exchange earnings [1]. In terms of small ruminants, Ethiopia ranks eighth in the world with an estimated number of 4 million sheep and 5.2 million goats [2]. These animals are an important asset for people with lower incomes and are exploited in the country for diverse purposes [3]. They also contribute 25% of domestically consumed meat, 50% of domestic needs in wool, 40% of skin, and 92% of the value of hide and skin exported to other countries [4]. However, due to the prevalence of peste des petits ruminants (PPR) and some related other diseases, the production and productivity were reduced by 50%–60% per year, causing direct and indirect losses [5].

Small ruminants affected by PPRV commonly show signs involving both the respiratory and digestive systems. Clinical signs include fever, thick discharge from the eyes and nose, painful mouth lesions, severe diarrhea, and pneumonia, which can ultimately result in death [6–8]. Its main transmission occurs through direct contact with the respiratory and digestive tract secretions from infected animals. It can cause high rates of morbidity and mortality, reaching up to 100% and over 90% in naïve herds, respectively. Mortality occurs between Days 5 and 10 after the infection, and recovering animals develop strong lifelong immunity [9].

PPR is one of the most important diseases affecting small ruminant productivity worldwide [10, 11]. It was one of the strongest limiting factors in the development of small ruminant farming in developing countries, where most people rely on sheep and goat production [12]. About 62.5% of the global small ruminants are at risk of PPR disease, which causes an estimated loss of 1.45–2.1 billion USD every year and threatens the small ruminant population worldwide [13, 14]. In Ethiopia, the mean flock‐level losses associated with PPR reached Ethiopian Birr 7485 (US$ 315) per affected flock [15].

Studies that have been undertaken on PPR so far have revealed the seroprevalence ranging from 2.1% in the Bench Maji and Kafa Zone of southern Ethiopia to 75.7% in Benishangul‐Gumuz [16, 17]. In Ethiopia, the lack of strict rules and regulations governing animal movement and certification is one of the main factors contributing to the spread of PPR along the small ruminant value chain. In addition, gaps in coverage of vaccination are other delaying factors for controlling PPR disease. Despite the existing disease challenges, farmers in the current study areas favor raising sheep and goats. Their remarkable adaptability to the environment, dynamic feeding behavior, and rapid reproduction cycle make them resilient and attractive choices. The outbreak of the disease was still being reported based on clinical signs. There is a limitation in effectively addressing and investigating PPR outbreaks and detecting PPRV through advanced molecular methods. Investigating PPRV seroprevalence and molecular profiles in sheep and goats is crucial for scientifically informed disease management and economic loss mitigation in Ethiopia’s small ruminant agriculture. Therefore, this study was conducted to detect PPRV and estimate the seroprevalence of PPR and associated risk factors that predispose to the disease in central Ethiopia.

## 2. Materials and Methods

### 2.1. Description of the Study Area

**FIGURE 1:**
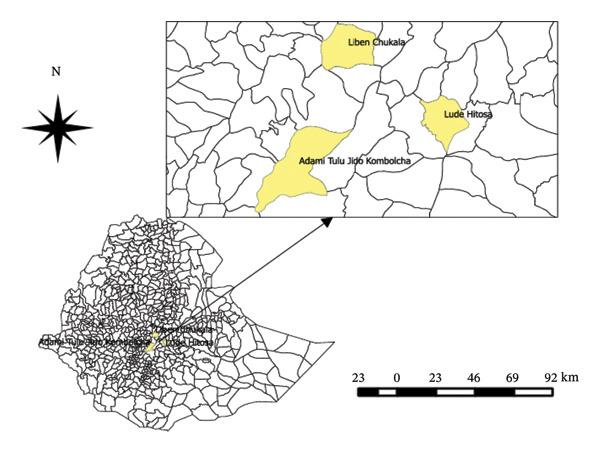
Geographical location of the study areas, developed from Ethiopian shape files using QGIS software.

The study was carried out in three different districts (Adami Tulu Jido Kombolcha and Liben Chukala District in the East Shewa Zone and Lude Hitosa District in East Arsi Zone) in central Ethiopia. A detailed description of the study areas, including geographic and climatic parameters, is presented in Table 1 and Figure 1 [1].

**TABLE 1:** Description of the study areas.

District	Altitude range (m)	Climate	Annual rainfall (mm)	Maximum temperature (°C)	Minimum temperature (°C)	Socioeconomic factors
Adami Tulu Jido Kombolcha	1600–2550	Tropical	600–900	27	15	Primarily agrarian economy; subsistence farming, livestock rearing; limited access to healthcare, education, infrastructure
Liben Chukala	1500–3200	Subtropical	1200	23	10	Agriculture‐based economy; maize, teff, barley cultivation, livestock rearing; access to markets, veterinary services, agricultural inputs
Lude Hitosa	1200–3100	Tropical	800–1000	28	16	Agriculture‐driven economy; wheat, barley, pulses cultivation, dairy and meat production; land tenure, access to water, market opportunities

### 2.2. Study Design, Study Population, and Clinical Investigation

A cross‐sectional study was conducted from December 2021 to July 2022 to investigate an outbreak of PPRV. Regional laboratories and district‐level animal health professionals reported outbreaks. Small ruminants were grouped by species (ovine and caprine), sex (male and female), and age (young and adult), as reflected in the results. Animals older than 6 months (to avoid maternal immunity) were included in the study for the serological study of the disease [18], while all age groups showing clinical signs of PPRV were considered for viral detection. During the outbreak investigation, 250 small ruminants were examined physically, and 25 of them with typical clinical signs of the disease were purposively sampled for viral isolation.

### 2.3. Sample Size Determination

Of 250 suspected small ruminants, 25 were sampled for isolation and molecular detection of the PPR virus (PPRV). The sample size for the collection of sera for the seroprevalence study was determined using a formula as described by Thrusfield (2018) [19]:

(1) N= Z2×pexp1−pexpd2,

where *N* is the required sample size for a large population, *Z* ^2^ is the value of *z* at a 95% confidence interval (*Z* = 1.96), *p*exp is the expected prevalence, and *d* ^2^ is the desired absolute precision (marginal error  =  5%).

An expected seroprevalence of 48.5% [20] was used to calculate the required sample size, which was 384. Sample sizes were proportionally allocated to each species based on their population in the study areas.

### 2.4. Sampling Procedures

A multistage sampling approach was used to study the seroprevalence of PPRV antibodies. The study focused on Adami Tulu Jido Kombolcha and Lude Hitosa districts in the East Arsi and East Shewa Zones of central Ethiopia. Five kebeles were selected based on the proportional size of the small ruminant population. Sampling occurred across 13 selected peasant associations (PAs) until the required sample size was achieved, with 10% of small ruminants per group sampled using simple random sampling. Species, age, sex, recent introductions to herds, herd size, and addresses of sampled animals were considered to assess risk factors for disease exposure. Animals were categorized into two age groups based on their dental eruption status [21]. Household groups of small ruminants that existed together during sampling were classified into small (< 10), medium (10–30), and large (> 30) herd sizes [36]. Importantly, there was no evidence of PPR vaccination in the study area or adjacent zones, as verified through available records.

### 2.5. Sample Collection and Preparation

Paired swab and postmortem samples were collected from suspected PPR cases for viral isolation and characterization. The swab samples were placed into a cryovial containing 1.5 mL of virus transport medium (VTM) containing phosphate‐buffered solution (PBS), antibiotics, and an antifungal agent. The cryovials containing samples were labeled and placed in an icebox, and relevant information was recorded in the designated format.

In postmortem cases, tissues were carefully taken from organs with visible lesions suggestive of PPR using sterile scissors and forceps. The samples were transferred into sterile bottles containing 4 mL of VTM with antibiotics and antimycotic agents, as per the National Veterinary Institute (NVI) standard formulation, and transported on ice. Both tissue and swab samples were transported to the NVI of Ethiopia virology laboratory under standard conditions in an icebox and kept at −20°C until further use. Tissue specimens were minced with sterile scissors, ground under aseptic conditions with a mortar and pestle within a BSC II using sterile PBS to create a 10% (w/v) tissue suspension. Each suspension, consisting of 1 g of tissue mixed with 9 mL of PBS, was centrifuged at 3000 rpm for 20 min at 4°C. The resulting tissue supernatants were collected and stored at −20°C for testing [7].

Blood samples (4 mL) from the jugular vein of each animal were collected using a needle holder, 21‐G sterile Venoject needles (BD, Plymouth PL6 7BP, UK), and sterile Vacutainer tubes. Vacutainer tubes were labeled and left at room temperature for 24 h in a tilted position to collect serum. Afterward, the serum was transferred into labeled 2‐mL cryovials, coded by village PAs and district, with parameters for risk analysis. The cryovials were stored in the district veterinary clinic refrigerator. The serum was transported to the NVI, Ethiopia virology laboratory under standard conditions (on ice), and kept at −20°C until screened for antibodies against PPRV exposure using serological analysis [7].

### 2.6. Molecular Detection of the Virus

#### 2.6.1. Sample Processing and RNA Extraction

Swab samples were thawed and vortexed, and then, the suspensions were clarified by low‐speed centrifugation at 3000 rpm for 20 min. The supernatant (1 mL) from each sample was collected and placed into labeled cryovials. Viral RNA was extracted using the QIAamp Viral RNA Mini Kit (Qiagen, Germany) according to the manufacturer’s instructions.

#### 2.6.2. Reverse Transcriptase Polymerase Chain Reaction (RT–PCR) for Amplification

Initially, RNA was extracted from sample suspensions by using the QIAamp Viral RNA Mini Kit (Qiagen, Germany). The one‐step RT–PCR was conducted by using the QIAGEN Kit, Germany (the Qiagen One‐step RT–PCR kit) as per the manufacturer’s instructions. The reaction mix contained the following reagents: 4 µL of RNase‐free water, 5 µL of 5X PCR buffer (1X), 2.5 mM Mg^2+^, 5 µL of Q‐solution, 1 µL of dNTPs, 1 μL of one‐step RT–PCR enzyme mix, containing reverse transcriptase and thermostable DNA polymerase (Taq DNA polymerase), 2 μL NP3 (forward primer), and 2 μL NP4 (reverse primer). Each primer of PPR NP3 Forward (5 pm/μL; 5’‐TCTCGGAAATCGCCTCACTAC‐3’) and PPR NP4 Reverse (5 pm/μL; 5’‐ CCTCCTCCTGGTCCTCCAAGACTG‐3’) was used to amplify a 351‐bp fragment of the PPRV nucleocapsid gene [22].

Amplification was done by one‐step RT–PCR with the final reaction volume of 25 μL, containing 20 μL of the prepared master mix and 5 μL of RNA template in an Applied Biosystems 2700/2720 thermal cycler. The reverse transcription and PCR were carried out sequentially in the same tube. The RNA obtained was converted to cDNA using a reverse transcriptase enzyme. The cDNA was amplified using PPRV‐specific NP3 and NP4 primers [23]. Both reactions were run under the same thermal profile: reverse transcription at 50°C for 30 min and initial denaturation at 95°C for 15 min (1 cycle), followed by 35 cycles of denaturation at 94°C for 30 s, annealing at 55°C for 30 s, and extension at 72°C for 30 s, with a final extension at 72°C for 5 min (1 cycle) using an Applied Biosystems 2700/2720 thermal cycler.

#### 2.6.3. Gel Electrophoresis and Visualization

RT–PCR products were separated by electrophoresis. A 2% agarose powder was mixed with 100 mL of a solution containing 98 mL of distilled water and 2 mL of 1X TAE for running the reaction. The mixture was boiled for about 4 min and poured into a casting dish after the addition of 5 μL of intercalating dye to the boiled 1X TAE with 2% of agarose powder. By doing so, the comb was inserted on one side of the casting dish, which was manually prepared with plastics and removed, along with the inserted comb, after solidifying the gel after 20 min. The removed line of the comb makes a hole (well). Finally, the electrophoresis tank containing the solidified gel was filled with 1X TAE.

The PCR product within the PCR‐Rxn tube was vortexed, and a total of 10 μL (6 μL of PCR product and 4 μL of loading dye [Promega, Madison, USA]) was mixed by pipetting with a micropipette in a new tube and transferred to each well on the solidified gel filled with 1X TAE. A 10‐µL molecular ladder (Promega, Madison, USA), starting at 100 bp, was inserted at two ends and finally run on the gel electrophoresis at 120 V/100 mA for 1 h. The PCR products were visualized and imaged under UV light using a gel documentation system. A sample was considered positive for PPRV when a 351‐bp band was seen.

#### 2.6.4. Virus Isolation and Identification

##### 2.6.4.1. Cell Line, Culture Medium Preparation, and Monolayer Formation

For isolating the virus from clinical samples, the Vero cell line (CCL‐81), P‐31 was used [7]. This well‐described cell line is used because of its high susceptibility to PPRV, and designating the passage makes it transparent, without jeopardizing the validation of our results. A T‐75 cm^2^ flask with cultured Vero cells grown in Dulbecco’s modified Eagle’s medium (DMEM) was used for monolayer preparation. Before collecting the cells, the outside of the culture flask was cleaned with 70% ethanol. The culture medium was removed, and the Vero cell layer was washed with PBS containing antibiotics and an antifungal agent. Finally, 4 mL of trypsin was added to detach the cells. The entire process adhered to OIE protocols.

Following the addition of 2 mL of trypsin at 5% CO_2_ and a temperature of 37°C, the cell line was incubated for approximately 3 min. The flask was then removed from the incubator, and 10 mL of complete media (comprising 10% calf serum and 2% PBS) was added to the T‐75 cm^2^ flasks, mixed by pipetting. Subsequently, 30 mL of complete media was dispensed into T‐25 cm^2^ flasks. Another flask containing Vero cells maintained in complete medium without viral inoculation served as the negative control. The media with the Vero cell line culture was placed in the incubator for 24 h to establish a confluent monolayer, confirmed by observation under an inverted light microscope as evidence of Vero cell multiplication.

### 2.7. Viral Isolation

Positive samples from RT–PCR were thawed, homogenized, and, in the case of tissue specimens, diluted in 1X PBS. The mixture was then centrifuged at 3000 rpm for 20 min at 4°C. Subsequently, 1 mL of the supernatant was filtered using a 0.22‐μm pore‐sized nylon membrane syringe‐driven filter unit. The resulting filtrates were utilized as inoculum for virus isolation [24].

The medium, comprising 10% calf serum, was discarded from a fully covered layer that displayed evidence of Vero cell multiplication. The layer was then rinsed with PBS, which included antibiotics and an antifungal agent. A 1‐mL filtered supernatant was inoculated onto a T‐25 cm^2^ fully covered layer of Vero cells using the adsorption method and incubated for 1 h at 37°C with 5% CO_2_, intermittently shaken to facilitate virus adsorption. The control flask remained uninoculated, and after virus inoculum decantation, infected cells were washed with DMEM (serum‐free DMEM). Subsequently, 8 mL of maintenance medium (DMEM with 2% serum) was added, and the cells were incubated at 37°C for up to 6 days, with a change of maintenance medium every other day [25].

The inoculated cell cultures were observed daily under an inverted light microscope for the appearance of cytopathic effects (CPEs). Six‐day follow‐up assessed the presence of typical CPE, and in flasks without CPE development within this period, blind passages were executed using 1 mL suspension, which was added to a new 25‐cm^2^ flask of a confluent monolayer of Vero cells. Samples that did not show CPE after three blind passages were considered negative for virus isolation [23]. The supernatant of cultures that developed CPEs at one of the passages was harvested after 3 freeze–thaw cycles, and subsequently, the presence of the virus in the medium was confirmed by collecting and testing the cell culture supernatant by RT–PCR.

### 2.8. Serology

Collected serum samples were tested for PPR nucleoprotein (NP) antibodies using a competitive ELISA kit. A 0.5‐mL serum sample was transferred from the cryovial to a 96‐well plate, before being transferring into an ELISA microplate using a multichannel pipette. The test was performed according to the instructions of the manufacturer (ID Screen PPR competition, France) [24]. A monoclonal antibody (Mab)–based competitive ELISA was used for the detection of antibodies directed against the NP of the PPRV using a competitive ELISA kit. The microplates were precoated with purified recombinant PPR NP.

### 2.9. Data Management and Statistical Analysis

Field and laboratory data were analyzed using STATA Version 14.0. Laboratory results, expressed as percentages, facilitated comparisons of positive samples. Seroprevalence proportions were calculated based on fixed factors, and logistic regression analyses at a 95% confidence level were employed to assess the statistical differences in the effects of various risk factors. Variables with *p* < 0.25 and noncollinear attributes were further analyzed using multivariate logistic regression. The final model, developed through backward elimination, underwent evaluation for goodness of fit using the Hosmer and Lemeshow method. Confounding effects were assessed by examining changes in the odds ratio, and variables altering the estimated risk’s odds ratio by 20% or more were considered confounders and included in the model.

## 3. Results

### 3.1. Investigation of PPR Based on Clinical Manifestation

Outbreaks of PPR occurred in Huruta (Lude Hitosa district, East Arsi Zone) and Bachako (Liben Chukala district, East Shewa Zone) in central Ethiopia. Both sheep and goats in these areas exhibited typical clinical signs of PPR disease, including depression, abortion, matting of eyelids, diarrhea, coughing, sneezing, difficulty breathing with nasal and lacrimal discharges, congestion of the gum mucous membranes, and necrotic lesions on the lips. Pneumonic lungs, hemorrhages on the mucosal surfaces of the abomasum and large intestine, and inflamed lymph nodes were observed in postmortem examination.

#### 3.1.1. Genomic Detection

**FIGURE 2:**
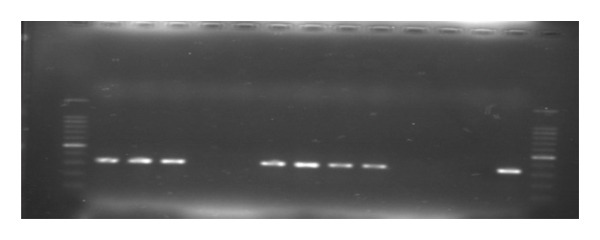
Agarose gel electrophoresis of PCR products (351 bp) amplified with NP3 and NP4 PPRV‐specific primers. Lane M: 100‐bp DNA molecular weight marker; Lane P: positive control; Lane N: negative control; Lanes 1–11: some field samples from Hurtua and Liben Chukala in East Arsi and East Shewa Zones in central Ethiopia.

Out of the 25 organ and swab samples subjected to RT–PCR using PPRV‐specific primers NP3 and NP4, which target the N gene of the virus, 10 samples (40%) tested positive for PPRV. These positive results were confirmed through gel electrophoresis of the PCR products, revealing an amplified fragment size of 351 bp. Samples exhibiting this specific fragment size (351 bp) were categorized as positive for the PPRV test, in comparison to the positive control (Figure 2).

In this study, 25 animals (9 sheep, 16 goats) were sampled. RT–PCR analysis showed 33.3% (3 out of 9) of sheep and a higher 47.75% (7 out of 16) positivity rate in goats for PPR. Overall, 40% (10 out of 25) of the animals tested positive. Importantly, goats exhibited a significantly higher prevalence of PPR compared to sheep.

The share of test positivity rate on RT–PCR with respect to sample type is presented in Table 2.

**TABLE 2:** RT–PCR test analyses for different PPRV suspected field samples.

Site	Sample type	No. of samples	RT–PCR positive (%)
Huruta	Nasal swab	10	4 (40)
	Tissue specimen	3	2 (66.6)
	Ocular swab	3	1 (33.3)
	Oral swab	2	0

Liben Chukala	Tissue specimen	2	2 (100)
	Nasal swab	2	1 (50)
	Ocular swab	2	0
	Oral swab	1	0

Overall		25	10 (40)

#### 3.1.2. Virus Isolation

**FIGURE 3:**
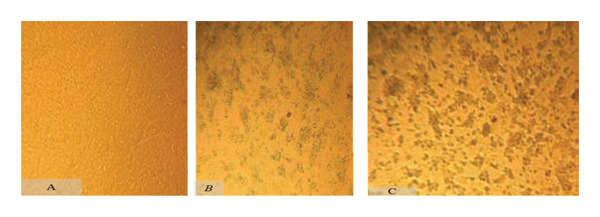
Photomicrographs showing cytopathic effects (CPEs) induced by PPRV in the Vero cell line. (A) Uninfected control showing a confluent monolayer of Vero cells at 3 days. (B, C) Infected cells exhibiting characteristic CPE, including cell rounding and syncytia formation at 4 days postinoculation.

Selected RT–PCR‐positive samples were successfully isolated in the Vero cell line. Four samples (two from tissue specimens and two from nasal swabs) exhibited CPE from the second to the fifth day postinoculation. Microscopic observations (Figure 3) reveal distinctive CPE in the Vero cell line. In Figure 3(B), on the fifth day postinoculation, cell rounding, aggregation, and spindle‐shaped cell appearance are evident. Figure 3(C), in the second passage, displays pronounced CPE, including rounding and syncytia formation, indicating successful virus recovery in the Vero cell line.

Out of the 10 RT–PCR‐positive samples, four exhibited viral growth on the Vero cell line. The presence of PPRV in the Vero cell culture was confirmed by RT–PCR analysis of the cell culture supernatant.

#### 3.1.3. Seroprevalence

Of 384 serum samples tested for PPRV antibodies using C‐ELISA, a total seroprevalence of 20.6% (79/384) was observed (Table 3). The distribution of positive results based on the origin of the sampled animals is detailed in Table 4.

**TABLE 3:** Overall seroprevalence of antibodies in sera of small ruminants using C‐ELISA in small ruminants.

Study areas	Tested samples	Positives	Percent (%)
East Shewa Zone	238	53	22.3
East Arsi Zone	146	26	17.8
Overall	384	79	20.6

**TABLE 4:** Proportions of seropositivity to PPRV via associated explanatory variables in small ruminants in Adami Tulu Jido Komibolch and Lude Hitosa districts (*N* = 384).

Variables	Categories	Frequency	Positive (%)
Sex	Male	189	34 (18)
	Female	195	45 (23.1)

Age	Young	149	20 (13.4)
	Adult	235	59 (25.1)

Species	Ovine	151	32 (21.1)
	Caprine	233	47 (20.2)

Herd size	Small	62	6 (9.7)
	Medium	145	22 (15.1)
	Large	177	51 (28.8)

RINA	Yes	159	45 (28.3)
	No	225	34 (15.1)

Herd composition	Mixed with cattle	210	28 (13.3)
	Unmixed	174	51 (29.3)

Origin	ATJK	238	53 (22.3)
	Lude Hitosa	146	26 (17.8)

*Note:* RINA = Recent introduction of new small ruminants into the preexisting small ruminants; ATJK = Adami Tulu Jido Kombolcha (district).

Abbreviation: CI = confidence interval.

The proportion of each explanatory variable’s seropositivity to PPRV is shown in Table 5.

**TABLE 5:** Univariate logistic regression analyses of assumed exposure variables associated with seropositivity of PPR risk factors (N = 384).

Variables	Categories	Tested	Positive (%)	95% CI	COR	95% CI	*p* value
Sex	Male (ref)	189	34 (18)	13–24	1.31	0.76–2.23	0.335
	Female	195	45 (23.1)	17–29			

Age	Young (ref)	149	20 (13.4)	8–19	2.12	1.17–2.81	0.013
	Adult	235	59 (25.1)	19–31			

Species	Ovine	151	32 (21.1)	15–28	1.31	0.75–2.23	0.340
	Caprine (ref)	233	47 (20.2)	15–25			

Herd size	Small	62	6 (9.7)	4–20	0.46	0.16–1.31	0.145
	Medium	145	22 (15.1)	10–22	0.69	0.35–1.39	0.305
	Large (ref)	177	51 (28.8)	22–35			

RINA	Yes (ref)	159	45 (28.3)	21–34	0.49	0.29–0.85	0.010
	No	225	34 (15.1)	11–19			

Herd composition	Mixed with cattle (ref)	210	28 (13.3)	9–18	1.84	0.93–3.62	0.077
	Unmixed	174	51 (29.3)	22–36			

District	ATJK (ref)	238	53 (22.3)	17–28	0.71	0.39–1.24	0.231
	Lude Hetosa	146	26 (17.8)	12–24			

*Note:* RINA = Recent introduction of new small ruminants into the preexisting herd; ATJK = Adami Tulu Jido Kombolcha (district); ref = reference variable that was statistically selected.

Abbreviations: CI = confidence interval, OR = odds ratio, SE = standard error.

### 3.2. Risk Factors

#### 3.2.1. Univariate Logistic Regression Analysis

Age, species, sex, herd size, history of recent introduction of new small ruminants, herd composition, and location of small ruminants were computed as associated risk factors of PPRV seropositivity in univariate logistic regression analysis (Table 6).

**TABLE 6:** Multivariable logistic regression analysis of potential risk factors in small ruminants among potentially associated risk factors.

Variables	AOR	95% CI	SE	*p* value
Age	2.21	1.26–3.93	0.65	0.006
RINA	0.51	0.31–0 0.85	0.13	0.011
Herd composition	2.51	1.48–4.27	0.67	0.001
_cons	0.04	0.01–0.22	0.03	0.001

*Note:* RINA = recent introduction of new small ruminants into preexisting small ruminants.

Abbreviations: CI = confidence interval, OR = odds ratio, SE = standard error.

#### 3.2.2. Multivariable Logistic Regression Analysis

Significant variables (*p* < 0.05) from univariable logistic regression were included in the final multivariable model. Multicollinearity analysis identified noncollinear categorical variables (e.g., age, herd composition, origin, small herd size, and history of recent introduction of new small ruminants), which were selected for multivariable logistic regression. Notably, herds with a history of recent introduction of new small ruminants are less likely to exhibit PPRV seropositivity (OR = 0.51), while age and herd composition are more likely to influence PPRV seroprevalence with odds ratios of 2.21 and 2.51, respectively (*p* < 0.05) (Table 6).

## 4. Discussions

A cross‐sectional study on sheep and goat populations detected PPR disease outbreaks in the study areas, as evidenced by clinical findings in suspected small ruminants displaying manifestations suggestive of PPR disease. The suggestive signs include fever, mucopurulent nasal and ocular discharge, lesions on the gums, diarrhea, coughing, depression, anorexia, and abortion. Congestion of the lungs, hemorrhages on mucosal surfaces of the abomasum, and inflamed lymph nodes were observed on postmortem examinations; in agreement with current clinical findings, the same clinical manifestations were reported [26, 27].

The current outbreak investigation revealed a higher prevalence of PPR in goats compared to sheep. This observation aligns with previous studies indicating that PPR is generally more severe in goats than in sheep [12], possibly due to PPRV inducing more pronounced immune suppression in goats [28]. Goats often exhibit more severe clinical manifestations, whereas sheep typically show milder symptoms or are asymptomatic [29]. However, the higher PPR prevalence in goats may reflect the larger proportion of goat samples collected in this study. Additionally, PPRV has been shown to inhibit leukocyte proliferation and induce apoptosis of peripheral blood mononuclear cells specifically in goats [30], although some studies suggest that both species can experience similarly high morbidity and mortality rates [31], possibly due to variations in PPRV virulence strains [32].

Among 25 samples tested by RT–PCR from suspected small ruminants during an outbreak investigation, 40% were positive for PPRV, indicating a lower prevalence compared to previous reports in various regions, such as Assosa Zone in Benishangul‐Gumuz Region, Ethiopia (45.4%) [33], and eastern Amhara in Ethiopia (46.4%) [34]. The current finding exceeds reported rates in Tanzania (29%) [35] and Algeria (Sahrawi Territories) (33.3%) [36]. Factors contributing to this disparity include variations in sample type, infection stage, RT–PCR gene targeting, viral load, the circulation of more virulent PPRV strains, and detection methods.

The isolates recovered at different passages; one isolate was recovered after a single blind passage on the fifth day. Two isolates were recovered during the second blind passage on the third and fourth day, whereas one isolate was recovered during the third blind passage on the second day of postinoculation. The same finding was also reported [38]. The analysis of the isolation results based on sample type indicates that the relative recovery of the virus using nasal swabs and tissue specimens from lung and intestinal mucosa is better than ocular, suggesting that nasal swabs are a better choice of sample for disease diagnosis [37, 38].

In the present study, some samples were positive by RT–PCR but did not recover on the cell culture line. These findings are consistent with the fact that some RT–PCR‐positive cases are negative for virus isolation [39]. The lack of viral growth in the cell culture may be due to the heat‐labile nature of the RNA virus, problems in maintaining the cold chain during sample transportation, low viral load in the clinical samples, and the type of cell culture used, which together may have prevented successful virus isolation [40].

Overall seroprevalence of this study (20.6%) is similar to the previous findings reported from the Somali (Dolo Odo) Ethiopian pastoral area (21%) [4]. In addition, consistent comparable similar seroprevalence of antibodies against PPRV has been documented in other Asian and African countries with an overall seroprevalence of 22.4% in Turkey [41], 22.1% in Tanzania [42], and 23.2% in Nigeria [43]. However, the current finding is lower than the findings of previous studies that reported 27.3% in Gambella [44], 29.2% in Siltie and Gurage [43], 30.2% in Oromia (Adama) [45], 32.1% in Borena Zone [46], 36.6% in Afar (Awash Fentale) [47], 40.2% in Afar (Adar and Mille) [48], and 75.7% in Benishangul‐Gumuz [18]. On the other hand, the current finding is higher than the study carried out in Benishangul‐Gumuz (Guba) with the seroprevalence of 8% [4]. These variations might be due to differences in agro‐climatic conditions, variations in production systems, cultural and social practices within different regions, management of animals, diagnostic tests, sampling procedures used, and levels of immunity [49, 50]. Moreover, this higher seroprevalence observed during an active outbreak of PPR could be attributed to the heightened exposure and transmission of the virus among susceptible animal populations.

Higher seroprevalence of PPR was recorded in adult small ruminants compared to young age groups. This finding is in agreement with the previous reports revealed by Assosa Zone, Benishangulgumuz Region [18], Siltie and Gurage Zones, Southern Ethiopia [43], and Bench Maji Zone, Southern Ethiopia [49], where they reported higher seroprevalence in adults. It has been documented that sheep and goats exposed to natural infection with PPRV at a very young age may carry antibodies for 1–2 years following exposure and remain positive for a long time [51, 52]. Moreover, age appears to be a risk factor for seropositive status, and this suggests that PPRV is highly immunogenic, with naturally infected animals remaining positive for a long time [53].

Small ruminants with a history of the introduction of new small ruminants were highly seropositive. This might be due to the introduction of new animals purchased from the live animal market, which were implicated as a source of PPR infection [54, 55]. Herd compositions consisting only of small ruminants showed higher seroprevalence (15.11%) than those kept with other livestock (13.33%). This suggests that other livestock have no role in the epidemiology of PPRV as they cannot serve as potential carriers and transmitters of the disease; thus, cattle are known to be a dead‐end host, and all attempts to induce clinical disease experimentally in adult cattle have failed [55].

The study on PPR outbreaks in small ruminants underscores the significance of considering potential unmeasured confounding variables. Regional agro‐climatic variations, diverse animal management practices, immunity levels, and cultural factors related to animal trade and movement are critical factors that may introduce biases in seroprevalence estimates. These variables could influence the observed associations between study variables, affecting the accuracy and interpretation of findings. Addressing these confounders through enhanced study designs and meticulous data collection methods is essential for ensuring the reliability of study outcomes and informing effective strategies for disease control and prevention.

## 5. Conclusion

Outbreak investigations and seroprevalence study findings indicated that PPRV was circulating in the current study areas. The presence of such a devastating virus poses a serious hindrance to small ruminant productivity; it is currently one of the major socioeconomic animal health problems in the area. A serological finding of this study provides preliminary information on PPRV seroprevalence and possible associated risk factors. The detection of antibodies through C‐ELISA in both administrative districts indicates widespread occurrence and endemic status of the disease in the study area due to natural infection; this negatively affects animal production and the socioeconomic status of farmers. Among the risk factors statistically associated with PPR exposure, the introduction of new small ruminants into the preexisting small ruminants, unmixed herd composition type, and being adult rather than young were significantly associated risk factors. The recommendations emphasize mixed raising of small ruminants with other livestock and implementing ongoing government efforts for PPR disease control in the study area. Caution is advised in interpreting findings of PPR outbreaks in small ruminants due to regional variations in agro‐climatic conditions and animal management practices. Further research, including molecular characterization of the circulating PPRV strain, is essential to validate findings across diverse socioeconomic and environmental contexts.

**Nomenclature**
CI: Confidence interval
CPE: Cytopathic effect
ELISA: Enzyme‐linked immunosorbent assay
KA: Kassaye Adamu
MS: Mishamo Sulayeman
NVI: National Veterinary Institute
OR: Odds ratio
PA: Peasant association
PPR: Peste des petits ruminants
PPRV: Peste des petits ruminants virus
TY: Teshome Yonas

## Author Contributions

T.Y. contributed to the conception of the research idea, designing, data collection, data analysis, interpretation of data, and writing and editing of the manuscript. M.S. contributed to the study concept, interpretation of data, and editing or reviewing of the manuscript. K.A. contributed to the conception of the research idea.

## Funding

The cost of this research work was covered by NVI.

## Disclosure

All authors read and approved the final manuscript.

## Ethics Statement

Ethical clearance for this study was obtained from the Animal Research Ethical Review Committee of NVI for collecting samples for the PPR disease with approved certificate reference number NVI/1529/5/, dated 3/11/2022.

## Consent

The authors have nothing to report.

## Conflicts of Interest

The authors declare no conflicts of interest.

## Data Availability

The datasets used and/or analyzed during the current study are available from the corresponding author upon reasonable request.
